# NiMoV and NiO-based catalysts for efficient solar-driven water splitting using thermally integrated photovoltaics in a scalable approach

**DOI:** 10.1016/j.isci.2020.101910

**Published:** 2020-12-09

**Authors:** İlknur Bayrak Pehlivan, Johan Oscarsson, Zhen Qiu, Lars Stolt, Marika Edoff, Tomas Edvinsson

**Affiliations:** 1 Department of Materials Science and Engineering, Solid State Physics, Uppsala University, Box 534, 75121 Uppsala, Sweden; 2 Solibro Research AB, Vallvägen 5, 75651 Uppsala, Sweden; 3 Department of Materials Science and Engineering, Solid State Electronics, Uppsala University, Box 534, 75121 Uppsala, Sweden

**Keywords:** Chemistry, Electrochemistry, Engineering, Materials Science, Energy Materials

## Abstract

In this work, a trimetallic NiMoV catalyst is developed for the hydrogen evolution reaction and characterized with respect to structure, valence, and elemental distribution. The overpotential to drive a 10 mA cm^−2^ current density is lowered from 94 to 78 mV versus reversible hydrogen electrode by introducing V into NiMo. A scalable stand-alone system for solar-driven water splitting was examined for a laboratory-scale device with 1.6 cm^2^ photovoltaic (PV) module area to an up-scaled device with 100 cm^2^ area. The NiMoV cathodic catalyst is combined with a NiO anode in alkaline electrolyzer unit thermally connected to synthesized (Ag,Cu) (In,Ga)Se_2_ ((A)CIGS) PV modules. Performance of 3- and 4-cell interconnected PV modules, electrolyzer, and hydrogen production of the PV electrolyzer are examined between 25°C and 50°C. The PV-electrolysis device having a 4-cell (A)CIGS under 100 mW cm^−2^ illumination and NiMoV-NiO electrolyzer shows 9.1% maximum and 8.5% averaged efficiency for 100 h operation.

## Highlights

• A new catalyst NiMoV is reported for the hydrogen evolution reaction
• A scalable thermally integrated PV-electrolyzer is designed for solar water splitting
• Interconnected PV-electrolyzer modules provide *STH* efficiency between 8 and 11%
• An upscaled CIGS-NiMoV-NiO device provides 8.5% *STH* for 100 h operation

## Graphical Abstract

**Figure:**
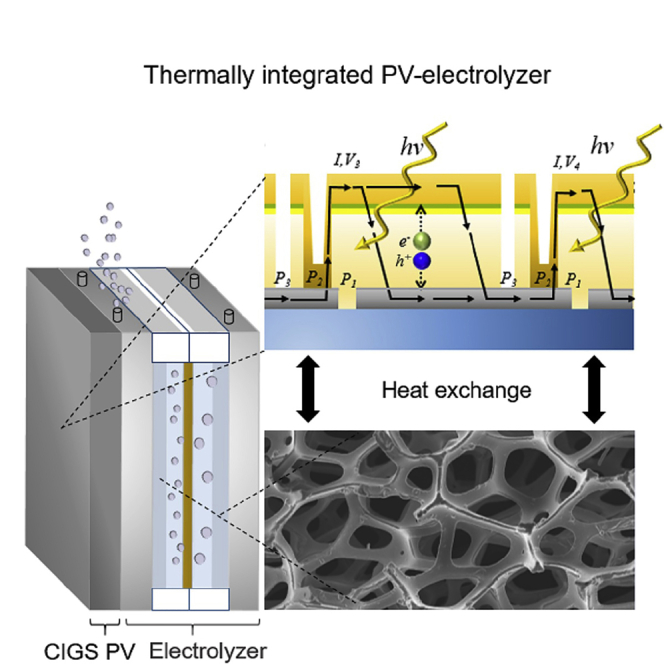

## Introduction

Solar hydrogen generators that combine capabilities of direct energy production and energy storage are attractive for sustainable energy generation technologies. A combination of water electrolysis and solar energy generators can be served for this purpose. The design of cost-optimized devices in terms of materials selection and process technology for photovoltaic (PV) devices, catalysts, and balance of system parts plays significant roles in the development of a viable hydrogen production system.

Alkaline electrolysis is the most widespread industrial water electrolysis technique (Leroy, 1983; Haug et al., 2017) with the advantage of having low cost, durability, and earth abundancy of the catalyst materials (Götz et al., 2016; Liu et al., 2017). Ni-based electrocatalysts have long been recognized as the most promising electrode materials due to their relatively high catalytic activity, abundancy, and low cost (Fan et al., 1994). NiMo alloys typically show superior catalytic performance for the hydrogen evolution reaction (HER) among the Ni-based alloys, which is attributed to a synergistic effect due to the electron-transfer between electron-rich Ni and electron-deficient Mo (Harinipriya and Sangaranarayanan, 2002). There are, however, several compelling reasons to look beyond bimetallic alloys and add a third metallic element to tune the electron-transfer in between the electron-rich Ni and electron-deficient elements, change the oxidation potential, and alter the corrosion stability of the catalysts. Specifically, it was shown that incorporation of NiMo with W provided better corrosion resistance, higher surface area, and better electrocatalytic properties toward HER as a result of synergetic effect of the three elements (Allam et al., 2018). Tafel slope and roughness factor of NiFeAl and NiFeMo trimetallic electrocatalyst was ∼30–35 mV dec^−1^ and 17, respectively, which was 54 mV dec^−1^ and 165 for a Ni electrocatalyst (Gerken et al., 2014). Density functional theory calculations also indicate that trimetallic systems are more beneficial than mono- or dimetallic systems for highly active catalysts (Cervantes-Gaxiola et al., 2013).

To provide an applicable system to produce solar hydrogen in practice, however, a full system needs to be constructed. Here, a directly coupled electrolyzer to the PV part can reduce losses from grid-losses and DC-DC conversion and enables a thermal exchange, which leads to simultaneous cooling of the PV and heating of the electrolyzer, which is beneficial for the total reaction (Bayrak Pehlivan et al., 2020). Integrated PV-electrolysis devices may therefore have advantages over wired PV-electrolysis due to the thermal exchange and as it can be constructed without inverters and longer wires, reducing Ohmic transport losses (Kirner et al., 2016; Welter et al., 2017). Till now, integrated PV-electrolysis systems have almost exclusively been made in small laboratory scale (Jacobsson et al., 2015; Bayrak Pehlivan et al., 2019) where the ability for upscaling is in important factor for the possibility for future implementation.

Here, we report the material properties and electrochemistry of a trimetallic NiMoV catalyst, the hydrogen production performance of thermally integrated PV-electrolysis devices combining (Ag,Cu) (In,Ga)Se_2_ ((A)CIGS) modules with a NiMoV (cathode) and NiO (anode)-based alkaline electrolyzer where the catalysts are made by scalable processes, and the materials are standard industrial materials used for sealing of thin-film solar cell modules.

## Results

**Figure 1:**
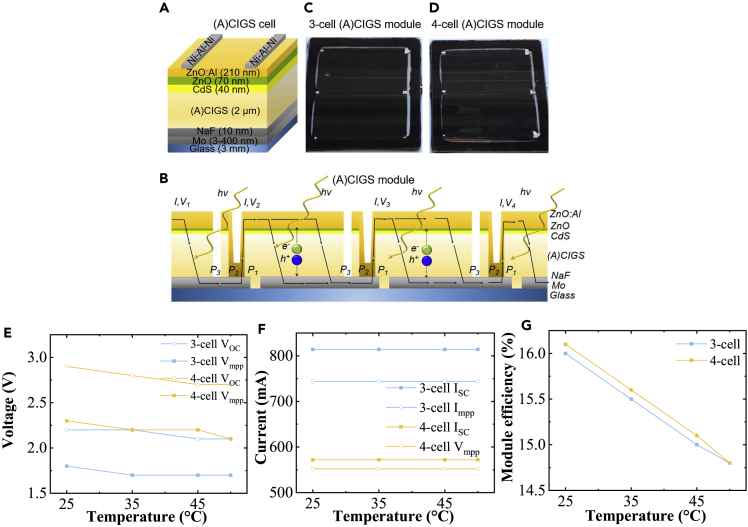
(A)CIGS cell, modules, and their PV parameters (A–G) (A and B) Schematic pictures of an (A)CIGS cell and a monolithically interconnected multi-cell (A)CIGS module, respectively. Layers not in relative scale. (C and D) Photographs, (E) open-circuit voltage (V_OC_) and voltage at maximum-power-point (V_mmp_), (F) short-circuit current (I_SC_) and current at maximum-power-point (I_mmp_), and (G) module efficiency of the 3-cell and 4-cell (A)CIGS modules.

The (A)CIGS material and subsequent solar cell modules were fabricated with the device structure shown in Figure 1A. The (A)CIGS technology uses a combination of laser- and mechanically scribed monolithic interconnects, which is one of the key processes for higher module efficiency. In this method, (A)CIGS modules are divided into smaller cells interconnected in series and thus reduce resistive losses. Figure 1B shows an interconnected 4-cell CIGS module. 3- and 4-cell monolithically interconnected A-CIGS modules are developed with an active area of 82 cm^2^ for 3-cell and 78 cm^2^ for 4-cell modules shown in Figures 1C and 1D, respectively. The total area was 100 cm^2^, and active area was used for the calculation of efficiency.

To assess the performance under full working conditions and to match the PV performance to the catalyst system, the photovoltage drop under increased temperatures needs to be quantified. Under 1 sun illumination (100 mW cm^−2^), open-circuit voltage (V_OC_) of the 3-cell (A)CIGS decreased from 2.2 to 2.1 V, and it dropped from 2.9 to 2.7 V for the 4-cell module with increasing temperature from 25°C to 50°C (Figure 1E). Similar trends were observed for the maximum-power-point voltage (V_mpp_), residing in the range of 1.8–1.7 V for the 3-cell modules and 2.3–2.1 V for the 4-cell modules. The current density values at short circuit (I_SC_) were 814 mA for the 3-cell and 572 mA for the 4-cell, while the maximum-power-points, (I_mpp_) 744 mA for 3-cell and 552 mA for 4-cell, were constant at different temperatures (Figures 1F and 1G).

**Figure 2:**
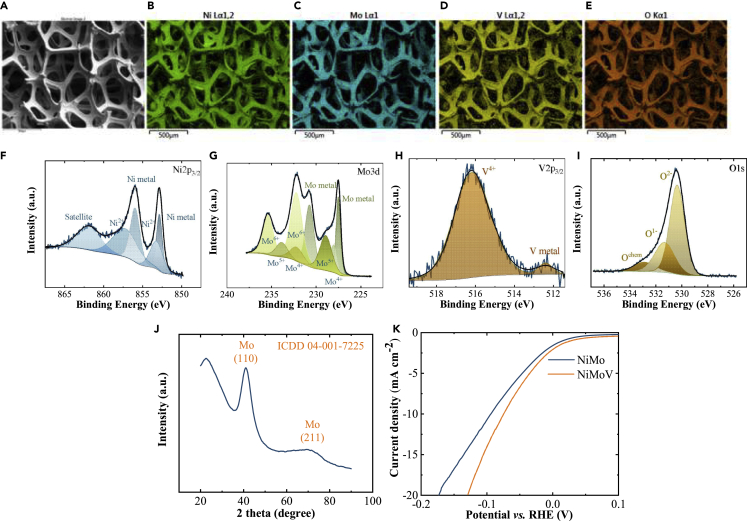
Material characterization and electrocatalytic property of NiMoV electrocatalyst (A–K) (A–E) SEM images and elemental mapping, (F–I) high-resolution XPS spectra, (J) XRD pattern for NiMoV thin film, and (K) linear sweep voltammetry measurement of NiMo and NiMoV catalysts in 1 M KOH with 5 mV s^−1^ scan rate for hydrogen evolution.

NiMoV electrocatalytic thin films were prepared by DC magnetron sputtering on Ni foam substrates with excellent uniformity according to scanning electron microscopy-energy dispersive X-ray spectroscopy (SEM-EDS) analysis (Figures 2A–2E). Details of the deposition can be found in Transparent methods. Surface and bulk composition of the NiMoV thin films were analyzed by EDS and X-ray photoelectron spectra (XPS). The relative ratio between the metal elements was found to be Ni(25)Mo(72)V(3) in the bulk with a slight Mo enrichment and V depletion at the surface with Ni(14)Mo(85)V(1). The XPS spectra with respect to Ni2p_3/2_, Mo3d, V2p_3/2_, and O1s are shown in Figures 2F–2I. All peaks were normalized with the C 1s peak set to 284.8 eV. The Ni 2p_3/2_ spectra were deconvoluted into four peaks, located at around 852.8, 853.2, 856.0, and 857.2 eV. The peaks at 852.8 and 853.2 eV can be assigned to Ni metal, whereas the peaks around 856.0 and 857.2 eV are characteristic for Ni^2+^ compounds (Biesinger et al., 2009). The peaks at 227.5 and 230.7 eV correspond to Mo metal for the Mo 3d spectra (Borgschulte et al., 2017; Khademi et al., 2009). In the V 2p_3/2_ spectra, two peaks at 512.4 and 516.2 eV match with V metal and V^4+^ states, respectively (Biesinger et al., 2010). For the O 1s spectra, the main peak around 530.4 eV is related to oxygen vacancy (Zhao et al., 2018). A peak at the binding energy of 531.3 eV has been proposed to be because of defective sites within the oxide crystal (Norton et al., 1977), adsorbed oxygen (Benndorf et al., 1982), or hydroxide species (Carley et al., 1983). A small peak at 532.9 eV may be caused by adsorbed water or possibly adsorbed O_2_ (Biesinger et al., 2009). The X-ray diffraction (XRD) showed that NiMoV was X-ray amorphous (Figure 2J). The center of the broad peaks around 40 and 73° best matches with the diffraction pattern of (110) and (211) planes for cubic molybdenum (ICDD 04-001-7225).

Figure 2K shows a comparison of electrocatalytic activity of NiMoV and NiMo thin films for hydrogen evaluation reaction. The required overpotentials, calculated without iR-correction, to drive a catalytic current density of 10 mA cm^−2^ were 94 and 78 versus reversible hydrogen electrode for NiMo and NiMoV, respectively. Reported overpotentials of NiMo-based electrocatalysts for hydrogen evaluation are collected in Table 1. Our result on NiMo catalyst was consistent with the previously reported catalytic activity of the catalyst. NiMoV thin films showed enhanced cathodic catalytic activity over NiMo thin films.

**Table 1:** Collected overpotentials and corresponding current densities (*j*) for NiMo catalysts for hydrogen evaluation reaction

Catalyst	Electrolyte	Overpotential (mV)	j(mA cm^−2^)	Ref.
NiMoV/Ni foam	1 M KOH	78	10	This work
NiMo/Ni foam	1 M KOH	94	10	This work
NiMo/Ti	2 M KOH	70	20	(Mckone et al., 2013)
NiMo/Cu	1 M KOH	152	20	(Zhao et al., 2017)
NiMo/Cu	1 M NaOH	34^a^	20	(Wang et al., 2014)
NiMo/Ni foam	1 M KOH	30^a^	10	(Fang et al., 2016)
NiMo/Ni foam	6 M NaOH	178	100	(Zhang et al., 2015)
NiMo/Ni meshes	33% NaOH	100	20	(Krstajić et al., 2008)

a Values are reported from I-V data that do not go to zero current density at zero overpotential, and thus inherently contain a large amount of uncertainty.

**Figure 3:**
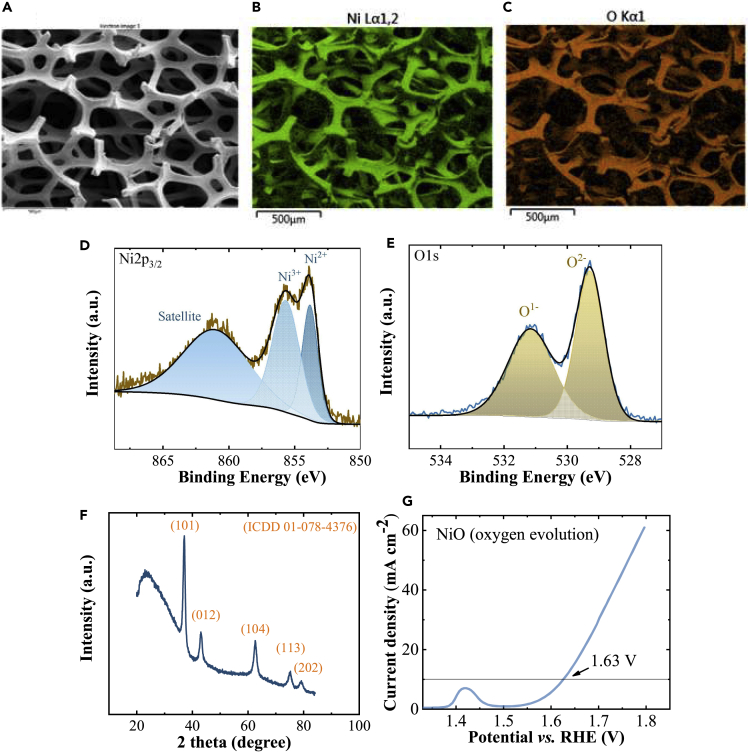
Material characterization and anodic electrocatalytic property of the NiO thin film (A–G) (A–C) SEM images and elemental mapping, (D–E) high-resolution XPS spectra, (F) XRD pattern for NiO thin film, and (G) linear sweep voltammetry measurement of NiO electrocatalyst in 1 M KOH with 5 mV s^−1^ scan rate for oxygen evaluation evolution.

An anodic NiO electrocatalyst is used as a counterelectrode to the NiMoV cathode. NiO thin films were homogeneously produced on Ni foams by the DC magnetron sputtering (Figures 3A–3C). The two peaks of Ni 2p3/2 spectra (Figure 3D) indicated that the NiO film contained Ni^2+^ (853.8 eV) and Ni^3+^ (855.5 eV). The peaks of O 1s spectra (Figure 3E) at 529.3 and 531.2 eV can be assigned to metal lattice oxide and defective oxide. XRD pattern of NiO had rhombohedral NiO phase (ICDD 01-078-4376) (Figure 3F). The overpotential for oxygen evaluation with a NiO film on Ni foam was 393 mV at 10 mAcm^−2^ in 1 M KOH at room temperature.

**Figure 4:**
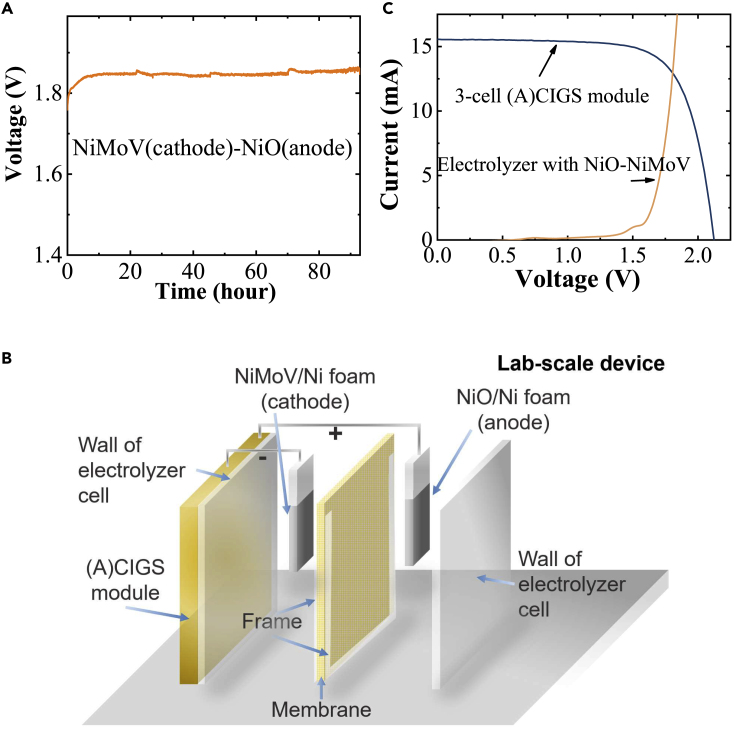
Solar-driven water splitting using a laboratory-scale (A)CIGS PV-NiMoV(cathode)-NiO(anode) electrolysis (A) Stability measurement of voltage versus time with a fixed current density of 10 mA cm^−2^ for NiMoV (cathode) and NiO (anode) with 0.25 cm^2^ catalyst area and 1.6 cm electrode distance without a membrane. (B) Schematic illustration of a laboratory-scale PV-electrolysis device. (C) Current-voltage measurements for a 1.6 cm^2^ 3-cell (A)CIGS mini module and an electrolyzer cell with 1.44 cm^2^ NiMoV (cathode) and NiO (anode) catalysts in the laboratory-scale device configuration.

Stability of the catalysis was investigated for a two-electrode system with NiMoV (cathode) and NiO (anode) thin film-coated Ni foams. Water splitting reaction was performed at 10 mA cm^2^ for >90 h. The average potential need was between 1.85 and 1.9 V (Figure 4A). After 90 h, the applied potential had increased by 0.5%. The origin of the periodic three small spikes around 23, 46, and 70 h was due to the electrolyte refilling.

A laboratory-scale PV-electrolysis device analogous to the upscaled thermally integrated device was formed for comparison. A schematic illustration of the assembled device is shown in Figure 4B. PV module and the electrolysis cell were in thermal contact. The anode and cathode of the electrolysis cell were placed in close contact with each other and separated by an anion exchange membrane. Thin glass was used as the wall of the cell to enable thermal coupling and heat exchange. Current-potential results of a 3-cell (A)CIGS mini-module with 1.6 cm^2^ area and a NiO(anode)-NiMoV(cathode) electrolyzer with area 1.44 cm^2^ and distance 2 cm between the electrodes are shown in Figure 4C. Solar-to-hydrogen (STH) efficiency of 10.0% was calculated from the intersection of current-voltage curves of (A)CIGS module with a PV efficiency of 14.5% and an electrolyzer that needs 1.8 V for 10 mA cm^−2^. The operating voltage can be expected to be 1.63 V for the combined PV-electrolyzer system (Figure 3G).

**Figure 5:**
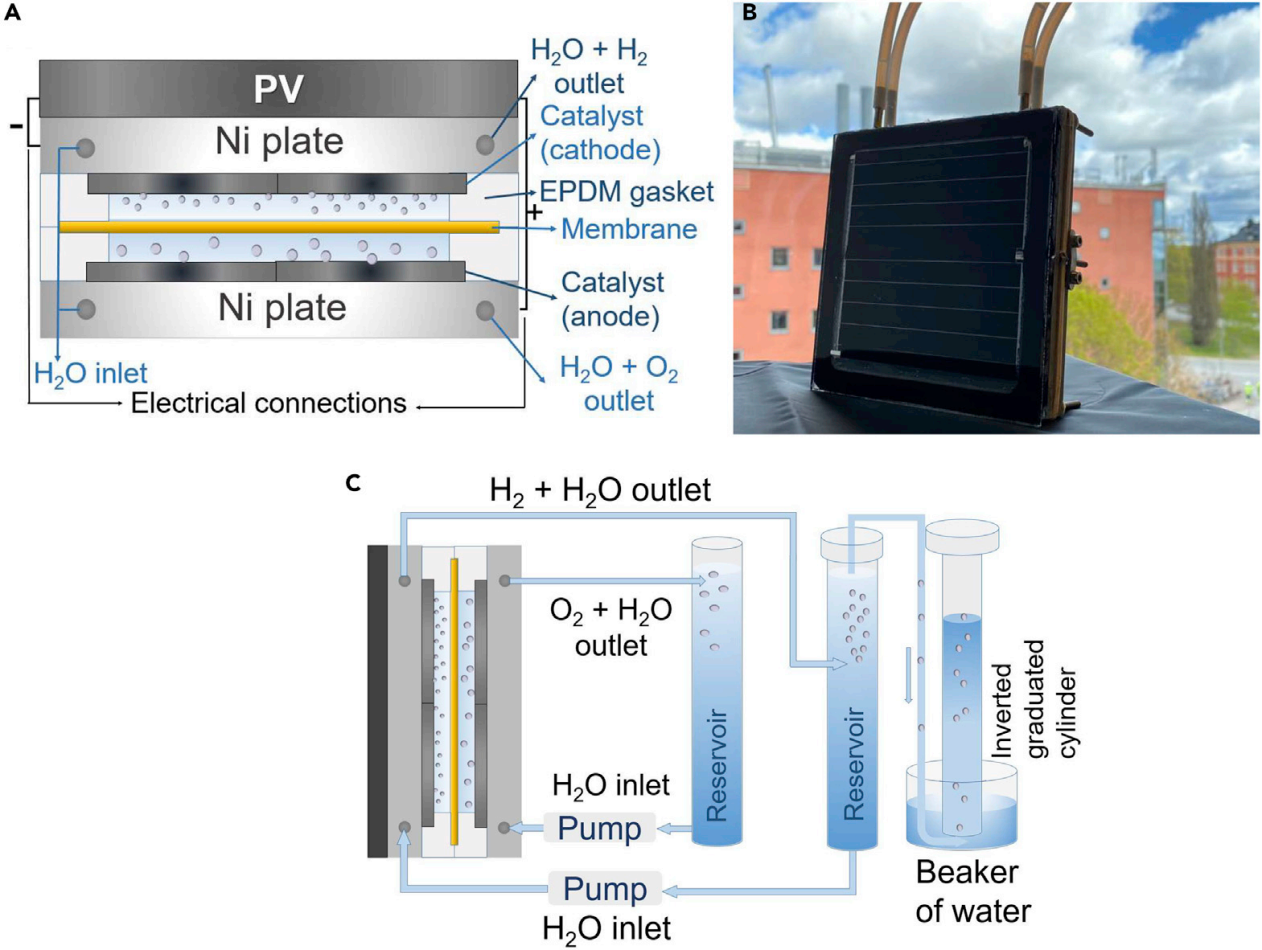
Thermally integrated PV-electrolysis device enlarged to 100 cm^2^ (A–C) (A) Schematic picture, (B) photograph of the integrated PV-electrolysis device, and (C) full setup for the gas volume measurements. Components are not in relative scale.

The possibility to transfer the materials and beneficial performance to larger scale is important and is performed in this study with sandwiched design allowing a close to zero gap in between the anode and cathode. A schematic device design and a photograph of the scalable thermally integrated PV-electrolyzer system are shown in Figures 5A and 5B, respectively. The electrolyzer is designed into a stack of Ni plate/cathodic catalyst/gasket/membrane/gasket/anodic catalyst/Ni plate. Ni plates (Ni 201 [99% Ni], and 130 × 130 × 5 mm) were used as the cover of the electrolyzer to minimize corrosion and thus provide electrolyzer components with long-term stability. For future cost or weight optimizations, the Ni-plates can be replaced with thinner foils or plastic covered with thin-film Ni. The active geometric area of the catalysts was 100 cm^2^. The gaskets were made from ethylene propylene diene monomer (EPDM) rubber with a thickness of 2 mm. An anion exchange membrane (Fumasep FAA-3-PK-130 Fuelcellstore) was used as the gas separation membrane. Screws in insulating bushings held the Ni plates together while compressing the EPDM gaskets to ensure the tightness of the electrolyzer. 3- and 4-cell (A)CIGS PV modules were developed and used for solar energy conversion. The PV and electrolyzer parts were glued together using boron nitride thermal paste to ensure thermal contact between them. The full setup for the gas volume measurements for the PV-electrolysis device is shown in Figure 5C. Two containers (polypropylene, 1 L) were filled with the electrolyte 1 M KOH (pH = 14). One bottle fed the anodic and the other fed the cathodic sides by pumping. Electrolyte circulation was done by the pumps through Teflon tubes with a flow rate of 50 mL min^−1^. All measurements were done with pumping. The volume of the gas was measured using inverted graduated cylinders.

**Figure 6:**
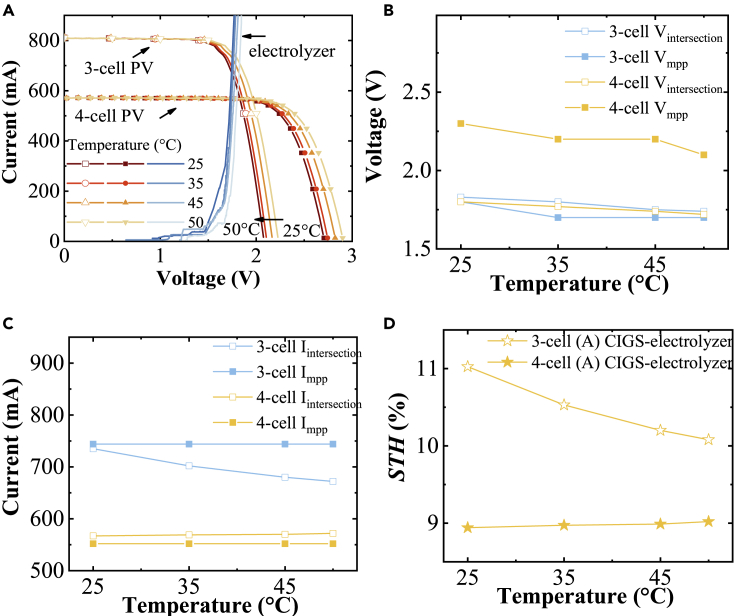
Current-voltage characteristics of the 3-cell (82 cm^2^ active area) and 4-cell (78 cm^2^ active area) (A)CIGS Modules and Electrolyzer Having NiMoV (Cathode)-NiO (Anode) (100 cm^2^ Catalyst Area) at Different Temperatures (A–D) (A) Current versus voltage, (B) voltage at maximum-power-point of the PV module curve (V_mmp_) and voltage at the intersection of the current-voltage curves of the PV module and the electrolyzer (V_intersection_), (C) I_mmp_ and I_intersection_, and (D) solar-to-hydrogen (STH) efficiency of the (A)CIGS PV-NiMoV-NiO electrolyzer calculated from the current at the intersection.

Before measuring the amount of produced hydrogen gas, current-voltage performance of the PV and electrolyzer parts of the upscaled integrated device were analyzed at different temperatures. To minimize the temperature fluctuation, the electrolyzer cell was mounted on one of the walls of the insulating box, whereas the pumps for electrolyte circulation were outside of the box and the tubes were thermally insulated. Heaters were placed in the box to control the temperature inside the box. The current-voltage measurements of the electrolyzer having NiMoV (cathode) and NiO (anode) electrocatalysts and 3- and 4-cell (A)CIGS module were done between the temperatures 25°C and 50°C (Figure 6A). Intersections for the integrated 3-cell (A)CIGS and the electrolyzer were on the high-voltage side of the maximum-power-point where the current changes more drastically with potential for PV systems. However, the intersection was on the plateau region for the 4-cell PV module, which gives a larger margin for light intensity and temperature changes and thus may lead to a higher yearly hydrogen yield.

The intersection voltage for the present catalyst system was much closer to the maximum-power-point voltage of the 3-cell module, whereas it was more comfortably placed for the 4-cell module (Figure 6B). The intersection current with changing temperatures for the 4-cell module is thus more stable and continuously placed at a lower potential than the maximum-power-point current of the 4-cell module when the temperature increased, in contrast to the 3-cell module (Figure 6C). These results are reflected in the efficiency variance of the complete PV-electrolysis devices as a more stable STH value around 9% for a 3-cell module and dropped from 11% to ∼10% with increased temperature (Figure 6D). In addition, comparison of the efficiency for the 3-cell laboratory-scale and upscaled PV-electrolysis devices, 10.0% STH efficiency of the laboratory-scale device with the PV efficiency of 14.5% (Figures 4C), and 11% STH of the upscaled device with 16% PV efficiency (Figure 6D) showed that the results have very good scalability, with retained high performance using the larger PV-electrolyzer device design.

**Figure 7:**
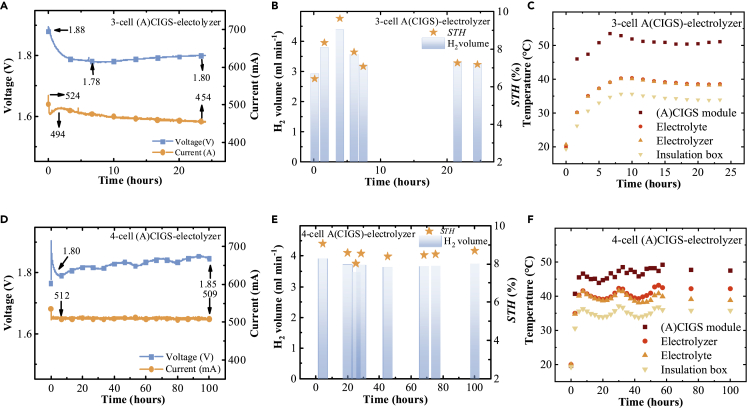
Solar-driven water splitting by the upscaled (A)CIGS PV Module and NiMoV-NiO Electrolyzer (A–F) (A and D) Monitored voltage and current, (B and E) hydrogen gas volume and STH efficiency for the thermally integrated PV-electrolysis devices, and (C and F) temperature recorded for the PV modules, electrolyzer, KOH reservoir electrolyte, and the insulating box. Data were collected for 24 and 100 h under 100 mW cm^−2^ illumination of 3-cell PV (82 cm^2^ active area)- and 4-cell PV (78 cm^2^ active area)-electrolysis (100 cm^2^ catalyst) devices, respectively.

Figures 7A and 7D show the monitored voltage and current during the gas volume measurement for the device with 3-cell and 4-cell (A)CIGS-NiMoV-NiO electrolyzer device, respectively. A higher stability of the operating current was obtained for the device with 4-cell (A)CIGS module as the intersections of the PV and electrolyzer curves fall in the plateau-current region of the current-voltage curve of the 4-cell PV. Figures 7B and 7E show the hydrogen generation rate and STH efficiency calculated from the hydrogen volume for 3- and 4-cell PV-electrolyzer devices, respectively. The average hydrogen generation rate was 3.5 mL min^−1^, which gives an average STH efficiency of 7.6% for the 3-cell PV-electrolyzer. For the thermally integrated 4-cell PV-electrolyzer device, the average hydrogen generation rate was 3.7 mL min^−1^ and the STH efficiency was showing a maximum and average of 9.1 and 8.5%, respectively.

Figures 7C and 7F show the temperature versus time for the (A)CIGS modules, electrolyzer, reservoir electrolyte, and insulation box. The temperatures fluctuated from a change in the temperature of the environment during the measurements days. For the 3-cell PV-electrolysis device measurements, the temperatures were stable after 10 h and average was 34°C, 38°C, 38°C, and 51°C for the box, electrolyzer, electrolyte, and PV module, respectively. For the 4-cell PV-electrolysis device, at the end of the 100-h test, the temperatures were 36°C, 38°C, 42°C, and 48°C for the insulation box, electrolysis, electrolyte, and PV, respectively. The reason for the increased STH in the initial 5 h and the decreased STH between 5 and 10 h can be attributed to the ambient temperature changes.

**Figure 8:**
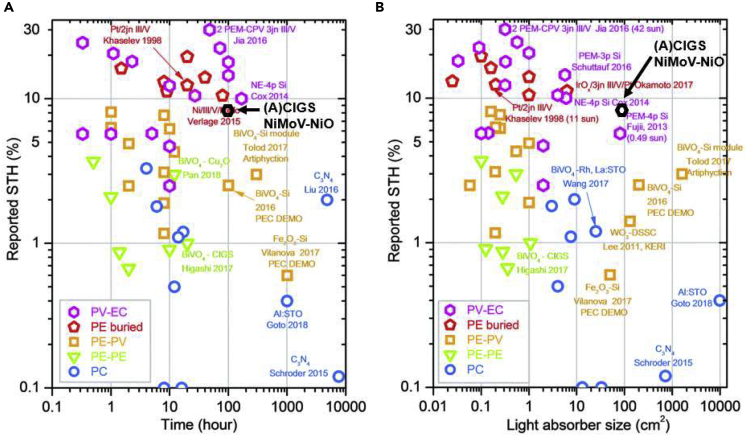
Comparison of the STHefficiency, stability, and scalability of the integrated 4-cell (A)CIGS PV-NiMoV-NiO Electrolyzer with Various Types of Solar Hydrogen Production Systems Reproduced from ref (Kim et al., 2019). with permission from the Royal Society of Chemistry, copyright 2020.c

A comparison of our results with reported literature data is shown in Figure 8 (Kim et al., 2019). The results show that the performance of the integrated PV-electrolyzer with 4-cell CIGS and NiMoV-NiO electrolyzer is among the largest integrated devices with efficiency around 9%–10% and high stability (100 h) (Figure 8A) and in the top-right corner with respect to *STH* efficiency versus size (Figure 8B) showing a promising scalability. Data with magenta-colored hexagon symbols are for PV-electrolysis approaches.

## Discussion and concluding remarks

In this work, a trimetallic cathodic catalyst, NiMoV, is developed using a scalable deposition method and applied in a thermally integrated PV-electrolysis device designed for solar-powered alkaline electrolysis. Elemental composition and X-ray spectroscopy were utilized to characterize the material, and a laboratory-scale (electrolyzer area 1.44 cm^2^) and an up-scaled (electrolyzer area 100 cm^2^) electrolyzer design are shown together with developed 3- and 4-cell ACIGS modules. The anodic and cathodic catalysts were positioned in close connection in the design to minimize mass transport limitations in the design, where modules of the size constructed here could be assembled into arrays for larger area installations for convenient maintenance and replacement of separate modules. The approach allows a heat transfer between the PV and electrolysis parts beneficial for improved system efficiency, where a decrease in PV efficiency from high operating temperature is compensated by increased electrolysis efficiency, and vice versa for low operating temperature. The scaled device has a size of 100 cm^2^, uses earth-abundant catalysts and standard sealing materials, and shows promising performance and stability. The efficiencies and stability are among the highest reported for integrated devices using non-concentrated light. Both catalytic current and gas volume measurement approaches were used for the determination of STH efficiency. The performance is lower than tandem PV approaches using concentrated solar light and precious catalysts (Liu et al., 2017; Tembhurne et al., 2019) but compares favorably with recent stand-alone water splitting approaches with smaller areas with 6.7% STH for a monolithic perovskite device (Liang et al., 2020) without precious catalysts and a recent CIGS-perovskite approach with precious catalyst showing 9% STH efficiency using a wired approach (Koo et al., 2020). Several of these approaches, however, are only shown for significantly smaller areas where the efficiencies may not be sustained upon up-scaling in cell or system size. The results in the present study instead bears promise toward large-scale implementations using matched integrated PV-electrolysis with stable thin-film PV materials, monolithic design, and utilization of earth-abundant catalyst components.

### Limitations of the study

Our work has reported a new and effective electrocatalyst NiMoV for both HERs, which was used in a thermally integrated PV-electrolyzer. Although we have achieved high catalytic performance here, an in-depth understanding of the catalytic process remains challenging. Therefore, soon, we will work on understanding the effect of V on the catalytic process for NiMoV catalysts with different V concentration. In this work, we reported *STH* efficiency for a newly designed thermally integrated PV-electrolyzer using (A)CIGS modules and thin-film catalysts in the electrolyzer. In the next step, new PV modules and nano-electrocatalyst will be produced to improve the operation point, and thus the *STH* efficiency.

### Resource availability

#### Lead contact

Further information and requests for resources should be directed to and fulfilled by the Lead Contact, Tomas Edvinsson (tomas.edvinsson@angstrom.uu.se).

#### Materials availability

This study did not generate new unique reagents.

#### Data and code availability

The published article includes all datasets/code generated or analyzed during this study.

## Methods

All methods can be found in the accompanying Transparent methods supplemental file.

## Supplemental information

Document S1. Transparent methods
