# Supplementary material for: NiMoV and NiO-based catalysts for efficient solar-driven water splitting using thermally integrated photovoltaics in a scalable approach
Source: iScience. 2020 Dec 9;24(1):101910. doi: 10.1016/j.isci.2020.101910 (PMC7758556; doi:10.1016/j.isci.2020.101910)
Supplement: Document S1. Transparent methods [file mmc1.pdf]

iScience, Volume *24*

Supplemental Information

NiMoV and NiO-based catalysts for efficient solar-driven water splitting using thermally integrated photovoltaics in a scalable approach

İlknur Bayrak Pehlivan, Johan Oscarsson, Zhen Qiu, Lars Stolt, Marika Edoff, and Tomas Edvinsson

Transparent Methods

PV preparation and characterization

The A-CIGS material and subsequent solar cell modules were fabricated on soda-lime glass substrates. The Mo layer was fabricated by DC magnetron sputtering, the 10 nm NaF layer was deposited by evaporation. The CdS layer was deposited by wet chemical bath deposition. A double layer of undoped ZnO and a layer of Al-doped ZnO were deposited by rf magnetron sputtering. A Ni-Al-Ni grid was evaporated through a shadow mask by electron gun evaporation. The A-CIGS layer was deposited by vacuum co-evaporation.

Three scribing steps called P1, P2 and P3 were used for the module processing. P1 was used for the back-contact forming the stripe-shaped molybdenum grid; P2 was used for the series interconnect formation between the adjacent cells; P3 was used for the neighbouring cell isolation after the top-contact deposition. The width of the cells was optimized to give appropriate voltage and current for running the catalysis at full scale, i.e. with PV modules with around 0.8 m^2 active area. Details of the CIGS module preparation can be found in the previous work (Bayrak et al., 2019).

Catalyst preparation and characterization

Thin films of electrocatalysts were prepared by DC magnetron sputtering coating on both sides of 25 cm^2 Ni foam substrates (thickness of 1.6 mm, the surface density of 350 g m^{-2}, and sheet resistance of 0.1 Ω/sq) using a Balzers UTT 400 reactive DC magnetron sputtering unit. The targets were a 5 cm diameter metallic discs with 99.99% purity from Plasmaterials. The distance of the target- to- substrate was 13 cm, and the substrate holder was rotated at 3 rpm for the homogeneity of the films. Pre-sputtering was performed for 5 min to clean the surface of the targets. The Ar flow rate was 50 ml min^{-1}, and the total gas pressure was kept at 30 mTorr for all depositions. The sputtering power of NiV (Ni (86 %) V (14 %)) and Mo targets were 120 and 180 W, respectively. NiMo was deposited at 120 W (Ni) and 180 W (Mo). Sputter deposition at a power of 200 W was carried out for NiO catalysts with 10 % oxygen-to-argon ratio. The film thicknesses on glass substrate determined by a Veeco Dektak 150 surface profilometry instrument were 165 ± 10 nm.

Surface morphology and homogeneity of the catalysts were analyzed by a Zeiss 1530 scanning electron microscopy (SEM)-Energy–dispersive X-ray spectroscopy (EDS) instrument operated using 5 kV electron accelerating voltage. X-ray mapping and elemental analysis were done by Aztec software.

X-ray photoelectron spectra (XPS) were used for surface analysis of the catalysts using a Quantera II (PHI, USA) spectrometer with monochromatic AlKα X-rays (hv = 1486.6 eV). The survey scans and high-resolution XPS spectra were measured using pass energies of 224 and 55 eV, respectively. The spectra were analyzed by the CasaXPS software.

X-ray diffraction (XRD) was performed with 1° of grazing angle using a grazing-incidence Siemens D5000 diffractometer with $CuK_{\alpha1}$ radiation at 1.5406 Å, 45 kV, and 40 mA. For the XRD analysis, the films were deposited on glass substrates.

Linear sweep voltammetry (LSV) measurements were performed using a CH Instrument model 760C workstation with a scan rate of 5 mV s^{-1}. The overpotentials of the catalysts were determined from LSV measurements of the catalysts (25 mm^2 areas in 1 M KOH) at 22 °C using Pt wire as counter electrode and Ag/AgCl (3 M KCl) as a reference electrode. Stability tests were performed in an electrochemical cell setup with a two-electrode configuration using a 5 mm distance between the anode and cathode and 25 mm^2 catalyst areas.

References

Bayrak Pehlivan, I., Edoff, M., Stolt, L., and Edvinsson, T. (2019). Optimum band gap energy of ((Ag),Cu)(InGa)Se_2 materials for combination with NiMo–NiO catalysts for thermally integrated solar-driven water splitting applications. Energies 12, 4064.
